# Albumin Infusion in Critically Ill COVID-19 Patients: Hemodilution and Anticoagulation

**DOI:** 10.3390/ijms22137126

**Published:** 2021-07-01

**Authors:** Giuliano Ramadori

**Affiliations:** Internal Medicine University Clinic, University of Göttingen, Göttingen, Germany Robert-Koch-Strasse 40, 37075 Göttingen, Germany; giulianoramadori@gmail.com

**Keywords:** COVID-19 hypercoagulability, hypoalbuminemia, lung injury kidney injury, ischemia, albumin infusion, fibrin, provisional clot, D-dimer

## Abstract

Hypercoagulation is one of the major risk factors for ICU treatment, mechanical ventilation, and death in critically ill patients infected with SARS-CoV-2. At the same time, hypoalbuminemia is one risk factor in such patients, independent of age and comorbidities. Especially in patients with severe SARS-CoV-2-infection, albumin infusion may be essential to improve hemodynamics and to reduce the plasma level of the main marker of thromboembolism, namely, the D-dimer plasma level, as suggested by a recent report. Albumin is responsible for 80% of the oncotic pressure in the vessels. This is necessary to keep enough water within the systemic circulatory system and for the maintenance of sufficient blood pressure, as well as for sufficient blood supply for vital organs like the brain, lungs, heart, and kidney. The liver reacts to a decrease in oncotic pressure with an increase in albumin synthesis. This is normally possible through the use of amino acids from the proteins introduced with the nutrients reaching the portal blood. If these are not sufficiently provided with the diet, amino acids are delivered to the liver from muscular proteins by systemic circulation. The liver is also the source of coagulation proteins, such as fibrinogen, fibronectin, and most of the v WF VIII, which are physiological components of the extracellular matrix of the vessel wall. While albumin is the main negative acute-phase protein, fibrinogen, fibronectin, and v WF VIII are positive acute-phase proteins. Acute illnesses cause the activation of defense mechanisms (acute-phase reaction) that may lead to an increase of fibrinolysis and an increase of plasma level of fibrinogen breakdown products, mainly fibrin and D-dimer. The measurement of the plasma level of the D-dimer has been used as a marker for venous thromboembolism, where a fourfold increase of the D-dimer plasma level was used as a negative prognostic marker in critically ill SARS-CoV-2 hospitalized patients. Increased fibrinolysis can take place in ischemic peripheral sites, where the mentioned coagulation proteins can become part of the provisional clot (e.g., in the lungs). Although critically ill SARS-CoV-2-infected patients are considered septic shock patients, albumin infusions have not been considered for hemodynamic resuscitation and as anticoagulants. The role of coagulation factors as provisional components of the extracellular matrix in case of generalized peripheral ischemia due to hypoalbuminemia and hypovolemia is discussed in this review.

## 1. Introduction

A recent publication drew attention to the effect of albumin infusion on hypercoagulability [D-dimer plasma levels, thrombosis-related complications, and death] in patients hospitalized because of SARS-CoV-2-infection [1]. Twenty-nine consecutive patients with COVID-19 PCR positivity, pneumonia, and a D-dimer plasma level above 1 microgram/mL and albumin serum levels < 3.5 g/dL were divided into two groups: 10 patients were treated with albumin infusions for 7 days, while 19 patients served as controls. Both groups received low-dose heparin. The mean age of the albumin group was 82 years, and in the control group, it was 73 years.

The amount of administered albumin was 80 g/day for the first three days, followed by 40 g/day for the other 4 days (400 g/week). There was an increase in albumin serum levels from 2.7 to 3.6 g/dL and a decrease in the D-dimer plasma level from 3.23 to 1.3 μg/mL. In the control group, the albumin serum level decreased from a mean value of 3.0 to 2.9 g/dL and the D-dimer plasma levels increased from 3.37 to 4.4 μg/mL. None of the patients were treated with corticosteroids but there was a higher number of the patients in the control group who were treated with piperacillin, tazobactam (16/19 vs. 6/10), and teicoplanin (5/10 vs. 15/19). Five patients (50%) in the albumin group were treated with the experimental antibody (Tocilizumab) against the interleukin-6 receptor vs. 13 (68.5%) of the control group. Four patients were treated with hydroxychloroquine in the albumin group and 15 in the control group, while 2 vs. 10 were treated with the antiviral lopinavir/Ritonavir.

The mean durations of hospital stays were 31 and 20 days for the albumin and control groups, respectively. One hemorrhagic event was observed in the albumin group, and two cases of ischemia, one pulmonary embolism, and one stroke were observed in the control group. While no fatal outcome was observed in the albumin group, four deaths were registered in the control group.

According to the authors, this small study suggests that albumin may exert an anticoagulatory activity compared to conventional preventive anticoagulation therapy with low dose heparin, which could not prevent the increase in the plasma level of D-dimer.

## 2. Albumin Is the Central Homeostatic Protein in Health and Disease

### 2.1. Physiology Including Response to Tissue Damage

Albumin is the main component of the non-corpuscular part of the blood and of the interstitial fluid. The two compartments are in close contact with each other. In fact, the interstitial albumin flows back into the serum through the lymphatic circulation with a turnover of 16 hours. Due to its molecular weight (68 kD) and molecular structure, albumin is the ideal protein to exert oncotic activity and to keep the necessary amount of water within the intravascular compartment [2].

Two factors are crucial for maintaining homeostasis and to ensure normal tissue oxygenation, namely, normal protein concentration and normal water intake.

The synthesis of albumin constitutes about 50% of the synthesized proteins in the liver and amounts to about 14–17 g/day. The regulation of this capacity is dependent on blood volume and vice versa. In fact, there is a physiologic increase in albumin synthesis up to the 20th year after birth [3,4]. On one hand, conditions that cause fluid loss induce a redistribution of albumin from the interstitium to the intravascular compartment, and on the other hand, the synthesis of albumin in the liver is increased [4] in the attempt to reabsorb water from the interstitium and from the tissues to maintain normal circulation in the vital organs, such as the kidneys, lungs, heart, and brain.

After the 20th year of age, a steady slow decrease of albumin synthesis in the liver begins. This is accompanied by a continuous reduction in blood volume [5,6]. The reason for this development is not exactly clear but the continuous increase of serum level of interleukin-6, the main cytokine of the acute-phase reaction, which accompanies the aging process [7], could contribute to the continuous inadvertent underhand “actualization“ of the body homeostasis. The solidity of the new equilibrium decreases continuously and the capacity of the “aging“ body to bear fluid deficit and to avoid a decrease in blood pressure and eventually loss of consciousness and death by “recruiting“ albumin and water from the interstitial compartment slowly fades away.

Dehydration may also be linked to hematocrit increase and to an increased risk of thrombogenesis [8].

Tissue damage is induced by acute disturbance of the integrity of the body by trauma, burning, and penetration and colonization of the body by infectious agents (mainly bacteria), which by quickly increasing in number, can lead to massive recruitment of granulocytes, mononuclear phagocytes, and lymphocytes.

Recruited cells release the main acute-phase mediators, such as interleukin-6, tumor necrosis factor alpha, interleukin-1, and gamma interferon.

The classical (physiologic) systemic reaction of disturbed homeostasis is characterized by an increase in body temperature up to 40 degrees Celsius some times lasting for several days, along with headache, somnolence, muscular pain, weakness, and loss of appetite [9,10,11].

The latter may be due to the direct influence of the cytokines on the brain [12], as well as to reduced motility of the bowel, which is also induced by the influence of the acute-phase cytokines [13]. Acute-phase reactions not only involve the upregulation of the secretory serum proteins, the acute-phase mediators also upregulate the synthesis of immunoglobulins against known foreign antigens from ordinary germs [14,15,16,17]. This may be the case of antibodies against the several coronaviruses that are responsible for the common cold [16,17].

Loss of wellbeing characterizes the sense of illness.

If the massive defense activity does not reach a quick success, the emergency mechanisms may not be able to maintain the minimal blood supply to all the organs of vital importance, such as the lungs, heart, brain, or kidneys, with the reduction in urine production being a measurable consequence. The reduced function of these vital organs [18,19,20,21] can be the true cause of death.

A systemic reaction can be triggered by a classical local reaction. When the damage is superficial in the skin or in a joint, rubor, dolor, calor, tumor, and functio laesa will be the observable signs. In many cases, however, bacteria can grow in the urinary tract, in the kidney, in the gallbladder, or in the lung (merely a few examples). Signs of systemic reduction of blood supply will be the more descrete characteristics of generalized ischemia and its consequence, namely, ischemic tissue damage. It has to be expected that the consequences of such processes are worse in older people [22], with chronic dehydration status and physiologically reduced blood volume. Preventive measures have to be taken to avoid hemodynamic emergency situations, which may even become irreversible.

Signs of acute illness may continue to be present even when the attacking noxae have already been eliminated. The kidney may be the organ that is most sensitive to dehydration-induced ischemia [19]. While diagnostic procedures are able to recognize the cause of acute large-vessel stroke, even in younger people [23,24], it is much more difficult to identify descrete clinical signs of subclinical ischemia due to dehydration at presentation when older patients complain of uncharacteristic cerebral or cardiac symptoms [20,21]. The detection of such possibly generalized descrete ischemic damage may become impossible when patients are transferred to the ICU, are mechanically ventilated, and discussions begin about appropriate antibiotic therapy because of mildly elevated inflammation markers, such as CRP, or about anticoagulants because of elevated serum plasma levels of D-dimers [23,24,25,26].

Laboratory investigations of the blood during an acute-phase situation show an elevated number of white cells [leukocytosis], mostly granulocytes; accelerated erythrosedimentation velocity; elevated serum levels of acute-phase proteins, such as C-reactive protein, serum amyloid A, fibrinogen, fibronectin v WF VIII, ceruloplasmin, alpha1 antitrypsin, lipocalin-2, lactoferrin, hepcidin, ferritin, and most of the complement proteins [9,10,11,12,13]. Most of the genes of the secretory proteins expressed in the liver are also expressed in extrahepatic sites and are also upregulated during the “emergency“ situation [12,27,28].

As mentioned above, the acute-phase cytokines also increase the production of gamma immunoglobulins (12–18% of the total serum proteins), mostly IgG, which are abundantly present in the serum, but also IgM, which may participate in increasing the phagocytic capacity of the tissue macrophages by binding the “intruders“ circulating in the blood with the help of circulating opsonins, mostly complement proteins.

At the same time, the tissue macrophages of the liver take up the infectious agents and the tissue debris, which have reached the systemic circulation, and eliminate them.

This may not be the case for some DNA-viruses, such as hepatitis B-virus, and for some RNA-viruses, such as hepatitis C-virus and HIV-virus, which cannot be definitively eliminated by the tissue macrophages after reaching the systemic circulation. This was convincingly demonstrated for HIV by Kirn’s group thirty years ago [29] on liver macrophages.

The enormous upgrading of the physiologic functions of a power plant and a sewage plant helps to explain the strong temporary increase in the liver volume observed under acute-phase conditions.

In fact, if the local and systemic defense apparatuses of the organism succeed in eliminating the damaging noxae and the damaged tissue, the liver activity returns to the conventional functions and the serum levels of the above-mentioned proteins return within their normal ranges [12,27,28].

### 2.2. Consequences of Hypoalbuminemia in Severe Diseases

If there is no additional damage as a consequence of systemic involvement [30], gene expression in the different organs [13,27,28] returns to the quo ante status.

The time to return to physical fitness (convalescence) may however be of variable duration and some of the laboratory changes, observed during the acute illness, may persist for longer periods and may be markers of hospital readmission [elevated CRP, D-dimer plasma levels, and hypoalbuminemia] [31,32,33,34,35,36,37,38,39].

The reason for this may lie in the fact that it takes weeks for the body to not only regain muscle volume [38,39] but also to produce the required amount of albumin, which had decreased during the disease, to reestablish the appropriate blood volume.

The reasons for the reduced synthesis of muscle proteins and albumin during the disease (before and during hospitalization) are mainly due to the reduced delivery of proteins with the diet, especially during mechanical ventilation, and less to the pre-translational downregulation of the synthesis capacity in the hepatocyte [40,41,42].

Therefore, the weakness that a patient feels in the recovery phase has three origins: the reduced muscle mass [38], which also involves the respiratory musculature [39]; the reduced albumin serum level [43]; and the consequently reduced blood volume, which produces a reduction in blood pressure.

For these reasons, death can occur in the convalescence phase up to one to five years after discharge from the hospital [44,45,46]. The cause of long-term consequences of the ICU stay may lie in the pre-ICU and/or prehospitalization phases [47]. Possible risk factors should be identified during the admittance of the patients in the hospital and then possibly checked histologically for plausibility [48,49,50]. In fact, risk factors should not only serve as prognostic markers [51,52,53,54,55,56] but they should possibly help to determine treatment procedures as in the case for the correction and monitoring of hypoalbuminemia [57].

The systemic symptoms and the laboratory changes may vary depending on the different attacking agents.

Viral infections cannot always be differentiated from bacterial infections based on the systemic clinical reaction when local pain is not present.

In fact, many viral infections can cause high fever, headache, somnolence, adynamia, loss of appetite, gastrointestinal disturbances, muscular pain, and weakness.

If the symptoms last for several days, the consequences for the blood circulation may be deleterious because of the massive loss of fluids and the massive reduction of the intake of nutrients, especially of proteins, with the diet [40,41].

The results of the laboratory studies of blood seldom show leukocytosis and an increase in serum levels of the classical acute-phase proteins, e.g., CRP or fibrinogen. Even if their serum levels are regularly increased, they seldom reach the magnitude observed during bacterial infections. The reduction of the blood volume and of the albumin serum level can, however, be as massive as in bacterial infections or even in complicatxed traumas.

Patients suffering from an unrecognized viral infection can be transferred from the emergency room to the ICU because of a hypovolemic shock, which quite often cannot be differentiated from a septic shock. This explains why, may be following the example of measles infection [58], in many cases, preventive administration of different antibiotics is started before any kind of blood volume measurement has been performed, as indicated by the increase in the amount of antibiotics administered [59] during the COVID pandemic. 

Hypoalbuminemia and dehydration can lead to damage of the function of vital organs, first of all, the kidney [60], which if not immediately corrected, can lead not only to shock but eventually also to death.

Viral infections can even precipitate hospitalization in patients with pre-existing frailty at a time when viral clearance has already taken place [47].

As mentioned above, albumin serum level and blood volume decrease continuously with increased age and hypoalbuminemia becomes a natural, additional risk of death for aged persons. However, this seems not to be the case for fibrinogen serum levels [61].

Hypoalbuminemia is a risk of death not only for all kinds of emergencies but also for elective surgical interventions [3] and is accompanied by hypovolemia. Untreated hypovolemia also leads to exaggerated inflammatory and immune responses that have an effect on tissues that are distant from the primary ischemic site.

The results are similar to the systemic inflammatory response syndrome and share many of the clinical features of bacterial sepsis [30].

Hypoalbuminemia is also a negative prognostic factor for all kinds of cancer treatments [62].

## 3. Albumin as a Life-Saving Drug: From the Second World War to the COVID-19-Pandemic

### 3.1. First Experiences of the Positive Effect of Albumin Infusion

The history of albumin treatment began during the Second World War [63] when fractionation of plasma proteins was performed. Albumin’s oncotic activity was measured and, following that, the consequences of albumin administration were determined. It was established that albumin’s single-chain structure with a molecular weight of 68 kD is ideal for exerting oncotic pressure in the blood vessels and to maintain elasticity in the interstitial compartment [63].

It was further calculated that each gram of infused albumin would hold 18 cc of fluid in the circulation by virtue of its colloid osmotic pressure and, therefore, 25 grams of albumin would represent the osmotic equivalent of 500 cc of citrated plasma. This amount of albumin was taken as the standard dose. A rapid decrease in hemoglobin concentration and hematocrit reading, indicating the transfer of extravascular fluid into the circulation, was observable as a consequence of an infusion of concentrated albumin.

This hemodilution effect, however, was not comparable to the effect induced by the administration of a saline solution. In fact, protein serum concentration increases d after albumin administration.

It was clear that patients with hypoproteinemia and edema caused by malnutrition or underfeeding because of emergency surgery needed up to 600 grams of albumin to recover, which was infused over several months in the hospital [63]. During hospitalization, it was clear that adequate protein intake is of importance to recover from hypoproteinemia and to maintain normal protein serum levels.

### 3.2. Albumin Infusion and Kidney Function in Cirrhotic Patients

Improvement of kidney function and an ascites reduction were observed when albumin was administered to patients with decompensated cirrhosis when diuretics failed to reduce ascites [64].

Ten years later, Losowsky and Atkinson clearly established that in order not to develop ascites, the albumin serum concentration has to be above 3.7 g/dL [65].

Although many patients with decompensated cirrhosis regained diuresis, their quality of life was greatly improved, survival was prolonged [observation time eight to nineteen months in 6 of 7 patients], and their ascites resolved after repeated albumin infusion [66]. Confirming previous experiences, the costs of that treatment became a major issue [67].

More or less at the same time, furosemide was introduced into clinical treatment and showed some additional success compared to the diuretics used before [68]. The use of albumin infusion for ascitic cirrhosis was definitively abandoned [69] despite the severe hypoalbuminemia.

A few years later, spontaneous bacterial peritonitis (SBP) was described. The resistance of ascites to diuretics was attributed to infection of the ascitic fluid. The number of granulocytes above 250 per microliter was and still is used as a surrogate marker for bacterial infection in the ascitic fluid [70].

Antibiotic administration was introduced into the treatment of SBP, sometimes even ex juvantibus, as some of the surrogate markers for SBP, such as CRP or procalcitonin serum level elevation, may be present in the absence of bacterial infection of the ascitic fluid. Even in the presence of severe hypoalbuminemia, albumin infusion was not part of the routine therapy of decompensated liver cirrhosis.

This changed after a prospective trial was performed [71]. It was shown that the combination of albumin infusion (1.5 g/kg body weight at days 1 and 3 after randomization) and antibiotics was superior to antibiotics alone for the treatment of kidney injury and spontaneous bacterial peritonitis in patients with decompensated liver cirrhosis and severe hypoalbuminemia resistant to diuretics.

Independent predictors of the development of renal impairment were found to be bilirubin and creatinine serum levels.

Albumin serum level as a risk factor was not mentioned.

An albumin infusion group was not considered in that study [71]. The measurement of blood volume was also not considered [72].

In a further study, the role of terlipressin as a vasoconstrictor in the treatment of patients with decompensated cirrhosis and hepatorenal syndrome was tested when albumin was administered together with terlipressin for a mean period of seven days.

The combination therapy albumin/terlipressin showed a dramatic therapeutic effect, with normalization of the creatinine serum level accompanied by increased diuresis. Increased natrium elimination in the urine and decreases in renin, aldosterone, and norepinephrine serum levels [73] were observed.

The first clinical trial of the nephroprotective effect of albumin infusion was achieved in patients with severe hypoalbuminemia, decompensated liver cirrhosis, and diuretic-induced fully reversible kidney injury [74].

Albumin infusion as a therapy for decompensated cirrhosis and severe hypoalbuminemia, even if largely used, is not yet included in the actual guidelines.

### 3.3. Albumin Infusion and the Influence of the Cost Factor

The confirmation of a study published 70 years ago, where several cases of long-term survival, under regular albumin infusion, were presented and the experience was repeated by Atkinson and Losowsky [65,66] and by Wilkinson and Sherlock [67] by injecting similar amounts of albumin. Atkinson and Losowsky also found that no patient with liver cirrhosis developed ascites when the albumin serum level was within the normal range (above 3.7 g/dL).

Wilkinson and Sherlock first mentioned that albumin infusion is a costly therapy in the introduction of their publication [67]

As previously mentioned, Gentilini et al. performed the first prospective study and demonstrated more than 20 years ago that long-term albumin infusion improves survival in patients with decompensated cirrhosis [74].

A further study, published by Caraceni et al. [75], clearly showed that long-term albumin infusion in patients with decompensated cirrhosis, with the albumin serum concentration kept at the level of 4.0 g/dL for 18 months, significantly prolongs survival, reproducing the observations published more than 75 years ago [64]. It was also confirmed that the prolonged administration of albumin also protected the patients against SBP, kidney injury and encephalopathy.

A similar experience concerning the need for long-term administration to reach a constant albumin serum level within the normal range was also previously made in a small group of 10 patients with decompensated cirrhosis and hypoalbuminemia this is resistant to diuretic therapy. It was clearly demonstrated that repeated daily infusions of 20 g albumin for 30 days were necessary to reach the elimination of ascites and reduction of the need for diuretic therapy. The total amount of albumin infused was about 600 grams [76], confirming the results published by Post et al., who needed 625 grams to maintain an albumin serum concentration at 4.0 g/dL [65]. This experience was routinely successfully repeated, as demonstrated in a case report of 75-year-old patients with diuretic-resistant decompensated cirrhosis as a further example [77]. The experiences showed that the reduction in kidney function is due to hypoalbuminemia and to the consequent reduction of blood volume. It also showed that the so-called kidney injury is fully reversible.

Furthermore, recent reports indicate that inflammation markers, together with increased interleukin-6 concentration in the serum and ascitic fluid, seem to correlate with higher creatinine serum levels and with severe hypoalbuminemia [78], suggesting that intestinal and systemic tissue ischemia may be the source of inflammation [78].

Meanwhile, albumin infusion in the first 24 h or in the first week was demonstrated to increase blood pressure in patients with shock, independent of the cause [79].

Although patients in that study had severe hypoalbuminemia, the albumin group only received 60 g of albumin during the observation time.

The authors did not find a survival difference between the patients in the albumin group compared with the patients in the crystalloid group regarding resuscitation therapy. They found, however, that the patients in the albumin group had a significantly lower heart rate and a significantly higher mean arterial pressure during the first 7 days than those in the crystalloid group. Those patients also had a higher albumin serum level. This further confirms that albumin induces an increase in blood volume.

Although it was known that this effect is of temporary duration when protein administration is not sufficient for the liver to synthesize enough albumin, the protocol was not changed.

Based on the previous experiences reported above, it was therefore not unexpected that the authors did not find any improvement in the 28-day survival of patients with shock.

However, in a correspondence paper to the same journal, Wiederman and Joannidis commented on the publication’s results after finding that albumin infusion increased survival in the subgroup of patients with sepsis [80]. Thompson [81] expressed the same opinion.

It has to be emphasized that in critically ill dehydrated patients, the measurement of blood volume should be performed routinely before fluid resuscitation and hemodynamic support therapy is started [28].

Furthermore, there should be no doubt that normal albumin serum levels (3.5–5.0 g/dL) have to be reached and maintained constant at that level to be sure that normal blood volume is guaranteed.

It is therefore not surprising that albumin is also essential for ameliorating the perfusion of the mucosa and the skin and to achieve optimal wound healing after surgery.

The high costs of albumin therapy are constantly mentioned in publications and are used to justify the restrictions imposed by the hospital authorities for the use of albumin as a drug in almost every country in the world.

## 4. Albumin, Dehydration, Inflammation, Hypercoagulation, and COVID-19

Hypercoagulation caused by dehydration is a well-known complication in critically ill patients. As mentioned before, dehydration can cause ischemia, which is then responsible for the serological signs of an acute-phase situation, such as an increase in CRP, procalcitonin, fibrinogen, and v WF VIII plasma levels [82]. In critically ill patients, hypercoagulation is diagnosed via measurement of D-dimer, fibrinogen, v WF VIII, the platelet number, and the prothrombin time.

Measurement of the D-dimer plasma level has been a diagnostic marker in cases of suspected venous vein thrombosis and pulmonary embolism [83,84,85,86], as the test measures the serum level of fibrin as a product of fibrinogen breakdown.

Venous thromboembolism prevention guidelines were also recently issued [87] but the measurement of albumin serum level was not mentioned.

D-dimer serum level measurement was introduced into the routine laboratory process of hospitalized COVID-19 patients early on as part of the patient’s characteristics in the first reports from China [56,88]. Hariyanto and coworkers [89] recently published the results of their meta-analysis about inflammatory and hematologic markers as predictors of severe outcomes in COVID-19 patients. Together with elevated procalcitonin, CRP, elevated D-dimer, and LDH levels significantly correlated with severe disease. Interestingly, an albumin serum level lower than 38.85 g/L was also a negative prognostic marker. In their discussion, the authors speculated about the increase in the D-dimer plasma level as a result of a dysregulated coagulation cascade with hyaline membrane formation at the level of the alveolar capillary [89].

**Figure 1:**
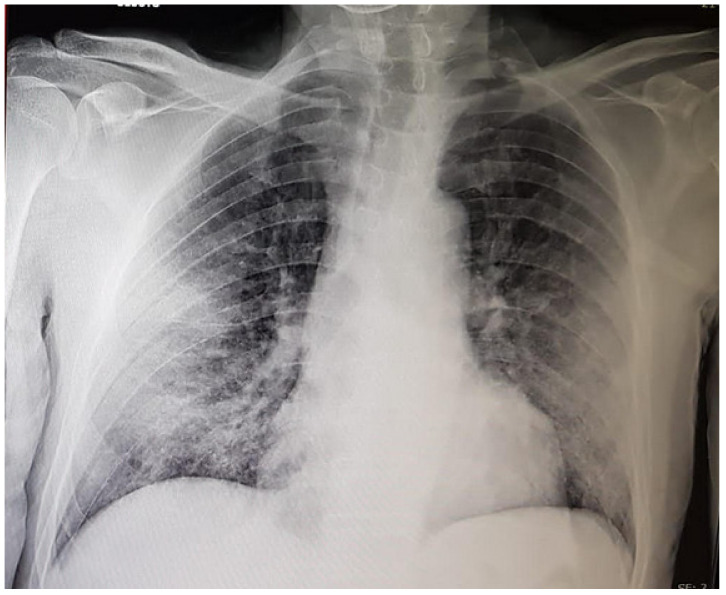
Conventional radiological investigation of the chest of a SARS-CoV-2-positive 65-year-old patient performed in the sitting position upon hospitalization. The D-dimer serum plasma level was slightly elevated [372 ng/mL DDU], fibrinogen was 975 mg/dL [180/380 normal range], the CRP serum level was 8.858 mg/dL, platelet number was 285 × 1.000/μL, prothrombin activity was 93% and INR 1.1, and the prothrombin time was 30 s. The leukocyte number was 10.000/μL and the lymphocyte number was within the normal range. The investigation detected bilateral ground-glass opacities with no signs of consolidation.

It has become a routinely determined marker in hospitalized SARS-CoV-2 patients with even early radiologic signs of pneumonia (Figure 1).

An increased serum plasma level of D-dimers became one of the risk factors for ICU transfer, mechanical ventilation, and death [90] when measured at admission in patients with SARS-CoV-2 infection.

It is also a marker for re-hospitalization and embolism after discharge from the hospital [44,45].

Two main routine methods are available, where the serum plasma levels are measured in ng/mL D-dimer unit (DDU; normal cutoff 250) or in μg/mL (normal cutoff 0.5 μg/mL fibrinogen equivalent unit (FEU)) [90].

In patients hospitalized because of COVID-19infection, hypercoagulation is a complication to be feared, although not all patients with increased serum plasma levels of D-dimer suffer from venous thrombosis and or embolic attacks [89,90]. Furthermore, an increase in fibrinogen, v WF VIII, and D-dimer serum plasma levels are observed in cases of inflammation [91,92]. The thrombosis risk seems to be increased when D-dimer plasma levels are four times higher than the cutoff value and the venous thrombosis screening is indicated [91,93].

It has to be emphasized, however, that fibrinogen plasma levels are increased and platelet counts stay in the normal range, even in COVID-19 patients with elevated D-dimer serum plasma levels [94,95,96,97]. This may mean that fibrin production may also take place in the absence of intravascular clot formation within the venous or, less often, the arterial vessels [98] and that D-dimer serum plasma levels may not be used as a surrogate marker for intravascular hypercoagulability or to adjust antithrombotic therapy [99].

Elevated D-dimer plasma levels could become an early sign of tissue ischemia.

There have been many hypotheses about the possible mechanisms that induce hypercoagulation in critically ill COVID-19 patients and about the special role of the endothelium in activating fibrin production from fibrinogen [98,99], which is not consistently associated with the presence of the virus [48,49,100]. Venous thrombosis prophylaxis strategies have been suggested for COVID-19 patients [101,102,103] and quite often an increase in dosage of the classical anticoagulants was initiated only based on the D-dimer-serum plasma levels but with no or little success [104].

On the other hand, thrombosis of the lung capillaries was quite often found without the presence of deep vein thrombosis [48,49] and the term primary pulmonary thrombi was suggested by Paramo [105].

Although in COVID-19 patients, the risk of thrombosis may persist even after discharge from the hospital [45,46,92] albumin serum concentration was seldom mentioned as a potential risk for thrombosis [57,92,106,107].

Albumin infusion in human septic shock seems to inhibit heparin-binding-protein-induced endothelial cell permeability [108], and through this mechanism, avoids kidney injury.

Violi et al. [1] showed that albumin serum levels on admission were strongly decreased in the 29 patients chosen for the study. The group of ten patients who were treated with albumin infusion had a mean albumin serum level of 2.7 g/dL compared to the 3.0 g/dL of the control group. The creatinine serum level was increased in both groups 1.2 mg/dL and 1.6 mg/dL in the albumin and the control group, respectively.

Although no blood volume measurements were performed, the decrease in the creatinine serum concentration in the albumin group strongly suggests that the beginning of kidney “injury” was of prerenal origin.

These findings not only confirmed the causal relationship between hypoalbuminemia and acute kidney injury but also that albumin infusion has a nephroprotective potential, as was recently underscored by Wiedermann [109].

Fibrinogen, fibronectin, and v WF VIII are three soluble components of both the coagulation system and the extracellular matrix. v WF VIII can be found along the endothelial layer of the veins and less often of the arterial capillaries [110].

It is possible that dehydration causes shrinkage of the endothelial cells in the different organs.

This may be of crucial importance in the thin vessels of the pulmonary alveolus. The reaction could be the attempt of the intravascular side to reduce the extravasation of fluid via the formation of a kind of provisional clot with a local deposition of fibrin, fibronectin, and v WF VIII. This could explain the fact that so-called hyaline material is detectable at the alveolar site of the capillaries of the lungs of COVID-19 patients [48,89,99,111]. Provisional clot formation with abundant fibrin deposition could also be the explanation for the recently reported findings of Blasco et al [112]. Authors found thrombi composed mostly of fibrin and some granulocytes in the material extracted from the coronary aspirates of five COVID-19 patients with ST-elevated myocardial infarction (STEMI). Only one of those five patients had a fourfold higher than normal serum plasma level of D-dimer, but a moderate presence of fibrin at the histological analysis.

Knittel et al. [113], Neubauer et al. [114], and Baruch et al. [115,116] studied the deposition of the three components—fibronectin, fibrinogen, and v WF VIII—in normal rat and normal human liver tissue, as well as in a model of tissue damage in the rat and damaged human livers [116]. Deposition of the three proteins can be shown in the wall of the central vein and of the portal vein of the normal rat and human livers. Acute tissue damage can lead to provisional clot formation with the deposition of fibrin, fibronectin, and v WF VIII in the damaged tissue (pericentral area) and the vessel wall.

The detectability of v WF VIII, which is exclusively synthesized and secreted by the endothelial cells, at least in the liver, is not limited to the endothelial cells of the large veins, but is also of the endothelial cells of the hepatic sinusoid, as indicated by the spot-like positivity within the liver parenchyma and in the cultured sinusoidal endothelial cells.

In the model of partial hepatectomy [115], an increase of the positivity for v WF VIII is detectable in the wall of the veins the liver of hepatectomized animals [compared to the veins in the normal liver ]. An increase in the serum level of v WF VIII was found not only in the blood of the hepatectomized but also in the blood of the sham-operated animals [white columns] indicating that the acute-phase reaction induced by the simple opening of the abdomen induced a release of the protein into the plasma. Von Willebrand factor VIII has to be considered as a main component of the circulatory homeostasis under normal and emergency conditions when the hydration status and serum albumin level are normal with a link to other proteins in the extracellular matrix [117].

In cases of hemoconcentration, hypernatremia can upregulate v WF VIII gene expression in the liver endothelial cells and support hypercoagulability [118], making it a risk factor for arterial and venous thrombosis [119].

Although other signs of dehydration are not reported in the patients, this consideration may have been one of the factors that encouraged the authors of the study to choose such a high dose albumin treatment during the first seven days after hospitalization.

Albumin administration induced a decrease in D-dimer plasma levels to almost a third of the baseline levels, while no change in the D-dimer plasma levels was observed in the control group. Four patients in this group died and ischemic complications were observed in four cases [1].

Administration of large amounts (400 g) of albumin in critically ill, old, and severely hypo-albuminemic SARS-CoV-2-positive patients within the first week of hospitalization is safe. In fact, no patients in the albumin group died and only one patient suffered a bleeding episode, which could be attributed to discoagulation as a side effect of albumin. No cardiac or pulmonary complications were observed. While the albumin serum levels in the albumin group reached the mean value of 3.6 g/dL, the albumin serum levels, which were also reduced at baseline in the control group, further decreased during the first week of hospitalization.

What can be learned from the study of Violi [1] and coworkers?

First and most importantly, the quantity of infused albumin should be high enough to increase the serum albumin concentration to the normal level. Second, albumin is then able to sufficiently increase the blood volume to protect the kidney, especially when potent diuretics are administered. This leads to an improvement in tissue perfusion not only in the kidney but also in all other organs, as indicated by the decrease of CRP serum levels.

Albumin seems to decrease the level of D-dimer in the plasma, not because of the hemodilution, but because of the reduction of the ischemic complications [28,109], which can be observed in different organs in patients with COVID-19 infection, experimental animals [113,114,115], and damaged human livers [116].

Endothelial cells of the liver are reported to also have the receptor for v WF VIII [120].

An important result from the publication of Violi and coworkers [1] is that the main mortality risk factor in COVID-19 patients, namely, hypoalbuminemia has to be taken care of immediately on admittance to the hospital by reestablishing normal serum levels.

Albumin infusion in association with conventional anti-aggregators may be the best anticoagulant therapy for critically ill patients with or without SARS-CoV-2 infection.
